# Triglycerides and leptin soluble receptor: Which one is the target to protect β-cells in patients with type 2 diabetes?

**DOI:** 10.3389/fendo.2023.1077678

**Published:** 2023-03-06

**Authors:** Hana Alzamil, Laila Aldokhi

**Affiliations:** Department of Physiology, College of Medicine, King Saud University, Riyadh, Saudi Arabia

**Keywords:** leptin soluble receptor, body composition, diabetes mellitus, HbA1c, triglycereides, insulin resistance

## Abstract

**Objectives:**

to study the relationships of leptin and leptin SR with adiposity indices, and glycemic indices in patients with type 2 diabetes mellitus (T2DM) compared to healthy subjects.

**Methods:**

This cross-sectional study involved 65 patients with T2DM and 63 healthy controls. Fasting plasma levels of leptin, leptin SR, insulin and lipid profile were measured by enzyme linked immunosorbent essay, basal insulin resistance and beta-cell function were assessed using the homeostasis model assessment.

**Results:**

leptin SR level was significantly higher in T2DM patients than in controls (5.8 ± 1.6 and 4.8 ± 1.3 respectively; p= 0.001). In patients with T2DM, leptin SR was negatively correlated with homeostasis model of β-cell function and body fat mass while it has a significant positive correlation with glycosylated hemoglobin (HbA1c). The independent predictors for leptin SR in patients with T2DM were triglycerides (TG) and HbA1c.

**Conclusions:**

elevated serum leptin SR level in patients with T2DM was positively correlated with TG and abnormal glucose metabolism which indicate that it plays a role in pathophysiology of T2DM. The association of elevated leptin SR level with high TG and deterioration of β-cell function indicate that in some individuals, particularly non-obese, dyslipidemia might be a cause rather than a complication of diabetes.

## Introduction

The hormone leptin is secreted mainly by adipose tissues and plays many important roles in the regulation of energy balance by suppressing the intake of food and stimulating thermogenesis, thus leading to loss of weight (1). Serum leptin levels are positively correlated with the percentage of body fat and body mass index (2). It has been suggested that obese people are resistant to the effects of endogenous leptin and even after administration of exogenous leptin there is no significant effect on weight loss (3). When leptin binds to its receptor in the hypothalamus, it stimulates many anorexigenic peptides and inhibit several orexigenic neuropeptides (4).

In humans’ plasma, leptin is found in bound, inactive form and free active forms. There is an equilibrium between the circulating binding protein and the free leptin. In lean subjects, most of serum leptin is bound to circulating binding proteins while in obese individuals the majority of circulating leptin remains free (5). Leptin acts by binding to leptin receptor (OB-R), which has a single transmembrane domain and belongs to class 1 cytokine receptor family. The shedding of OB-R extracellular domain produces the main binding protein for leptin in the blood, leptin SR. Animal studies and tissue culture experiments showed that an increased serum leptin SR was associated with inhibition of leptin signal transduction (6). Additionally, a study conducted in young populations found a relationship between serum leptin and its soluble binding protein levels on one hand with measures of adiposity and metabolic syndrome score on the other hand (7). Morioka and coworkers suggested that leptin SR is a factor that can affect pancreatic beta cells secretory functions in patients with T2DM (8). Additionally, Kang et al. found that triglycerides/glucose index can predict insulin resistance (9). Recently, a group of researchers reported that TG level can significantly predict the risk of developing prediabetes and diabetes (10). The present study aimed to investigate the levels of leptin, leptin SR and their correlation with lipid profile, obesity and glycemic control in patients with T2DM. We hypothesize that both leptin SR and TG have a profound effect on beta cell function and a complex interrelationship that needs further investigation.

## Methodology

This cross-sectional study enrolled 128 participants, 65 subjects were diabetic patients (34 males and 31 females) and 63 subjects were healthy controls (36 males and 27 females). The control subjects were healthy employees recruited by local advertisement and the diabetic patients were recruited from primary care clinics at King Khalid University Hospital, Riyadh, Saudi Arabia. This study was conducted at the Department of Physiology, College of medicine at King Saud University. The control group were evaluated using detailed history, clinical examination and investigations. Recruited patient were known to have T2DM for at least six months and the duration ranges between six months and 11 years. All patients with T2DM were receiving oral hypoglycemic drugs and only 18 (24%) patients were on lipid lowering agents. We excluded any patient with type 1 diabetes, acute infection, cardiovascular complications, nephropathy, neuropathy, amputation or those who needed admission. Patients and controls with pregnancy, using oral contraceptive pills or glucocorticoids were excluded.

The study protocol was approved by the ethical committee of institutional review board of college of medicine, King Saud University with approval number: 03/1342/R. All participants signed an informed consent and confidentiality was assured.

Body composition measurement obtained using the body composition analyzer (Biospace-InBody 3.0. SNBS 300504E 2003/04.272-Iyongieong-vi, yipjang-myeon, chanan-si, chungcheongnam-do, South Korea). The measurements included: body mass index (BMI), % body fat, lean body mass and waist-hip ratio (WHR). Before those measurements were taken, the subjects palms and soles were cleaned with electrolytes tissue, and information about their height, sex and age were fed to the machine. The subject was asked to stand with barefoot on the platform of the machine

Fasting venous blood samples were analyzed for blood glucose, HbA1c, basal insulin, lipid profile, leptin and leptin SR. Measurement of HbA1c was performed by Helena Glyco-Tek Affinity Column method, (Helena Biosciences, Europe, Colima Avenue, Sunderland Enterprise Park, Sunderland, Tyne & Wear, SR53 x B, UK). Lipid profile was estimated using Knonelab Itelligent Diagnostics Systems (Konelab Corporation, Ruukintie 18, FIN-02320 Espoo, Finland).

Insulin, leptin and leptin SR immunoassays were performed by quantitative standard sandwich ELISA technique using monoclonal antibody specific for these parameters with kits supplied by R&D Systems, (Abingdon, United Kingdom). The indices of basal insulin resistance and beta-cell function were assessed using the homeostasis model assessment (HOMA/IR and HOMA/B) in which HOMA/IR (mmol/L x µIU/mL) = fasting glucose (mmol/L) x fasting insulin (µIU/mL)/22.5 and HOMA/B = fasting insulin (µIU/mL) x 20/[fasting glucose (mmol/L) – 3.5].

For further analysis of the effect of obesity on leptin and leptin SR we subdivided the study group into four groups, non-obese control (n=30), non-obese diabetic (n=30), obese control (n=33) and obese diabetic (n=35). Obesity was defined as BMI more than 30 kg/m^2^ (according to WHO criteria). To test for the impact of glycemic control on leptin and leptin SR we subdivided patients into two groups, one with good control (HbA1c ≤ 7.5) which included 35 patients and the other with uncontrolled diabetes (HbA1c >7.5) represented by 30 patients.

## Statistical analysis

The data were analyzed by the computer software program Statistical Package for Social Sciences (SPSS version 20, Chicago). Descriptive characteristics and the lipid profile of the subjects were expressed as Mean± Standard Deviation (SD). Kolmogorov-Smirnov^a^ and Shapiro-Wilk tests were used to see that data is following normal distribution or not. Those parameters which were not following normal distribution were analyzed by non-parametric Mann Whitney test. For continuous data with normal distribution Student’s t-test was used. Correlations between leptin, leptin SR, HbA1c, BFM and markers of insulin resistance were determined by simple regression analysis. A stepwise linear regression model was constructed for leptin and leptin SR as dependent variables to find the independent predictors for these variables in patients with T2DM. Anova test was used to compare the level of leptin and leptin SL between four groups (control non-obese, diabetic non-obese, control obese and diabetic obese) then *post-hoc* test was performed.

## Results

Demographic characteristics, clinical features, insulin resistance indices and body composition for controls and T2DM patients were presented in **Table 1**. Age for control subjects ranges between 25 and 62 years (Mean: 47.22 ± 7.73) and for patients was 30-66 years (49.45 ± 10.2).

**Table 1:** Comparison of clinical characteristics, body composition and insulin resistance indices between healthy subjects and patients with T2DM.

Variables	Controls (n= 63)	Patients (n= 65)	P value
M/F	36/27	34/31	
Age (years)	47.22 ± 7.73	49.45 ± 10.2	0.790
BMI	28.9 ± 4.2	31.4 ± 5.7	0.005*
WHR	0.97 ± 0.07	1.03 ± 0.08	0.001*
SBP (mmHg)	117.3 ± 14.6	128.1 ± 16.8	0.001*
DBP (mmHg)	78.8 ± 9.8	80.6 ± 9.0	0.256
TC (mmol/L)	5.1 ± 0.8	5.0 ± 0.9	0.390
HDL (mmol/L)	1.2 ± 0.3	0.9 ± 0.2	0.001*
TG (mmol/L)	1.3 ± 0.7	1.9 ± 0.9	0.001*
FBG (mmol/L)	5.0 ± 0.5	7.9 ± 2.6	0.001*
LDL (mmol/L)	3.3 ± 0.7	3.2 ± 0.8	0.346
HbA1c (%)		7.3 ± 1.8	–
Fat Mass (kg)	26.6 ± 8.5	30.2 ± 10.6	0.040*
Body Fat %	34.9 ± 8.2	37.6 ± 7.4	0.032*
Basal Insulin uIU/ml	6.5 ± 3.3	10.5 ± 14.4	0.028*
Homa IR	1.5 ± 0.8	2.9 ± 2.4	0.010*
Homa B(%)	95.5 ± 61.3	48.6 ± 34.5	0.001*
Leptin ng/ml	30.6 ± 19.8	32.2 ± 19.5	0.331
Leptin SR ng/ml	4.8 ± 1.3	5.8 ± 1.6	0.001*

Values are expressed as mean ± SD, Insulin and leptin levels were compared by Mann.

Whitney test. All other parameters were compared by t test.

*P value significant when p≤ 0.05.

Leptin SR level was significantly higher in T2DM patients than in controls (5.8 ± 1.6 and 4.8 ± 1.3 respectively, P=0.001) while the difference was not significant for leptin (32.2 ± 19.5 and 30.6 ± 19.8 respectively, P=0.331).

**Figure 1:**
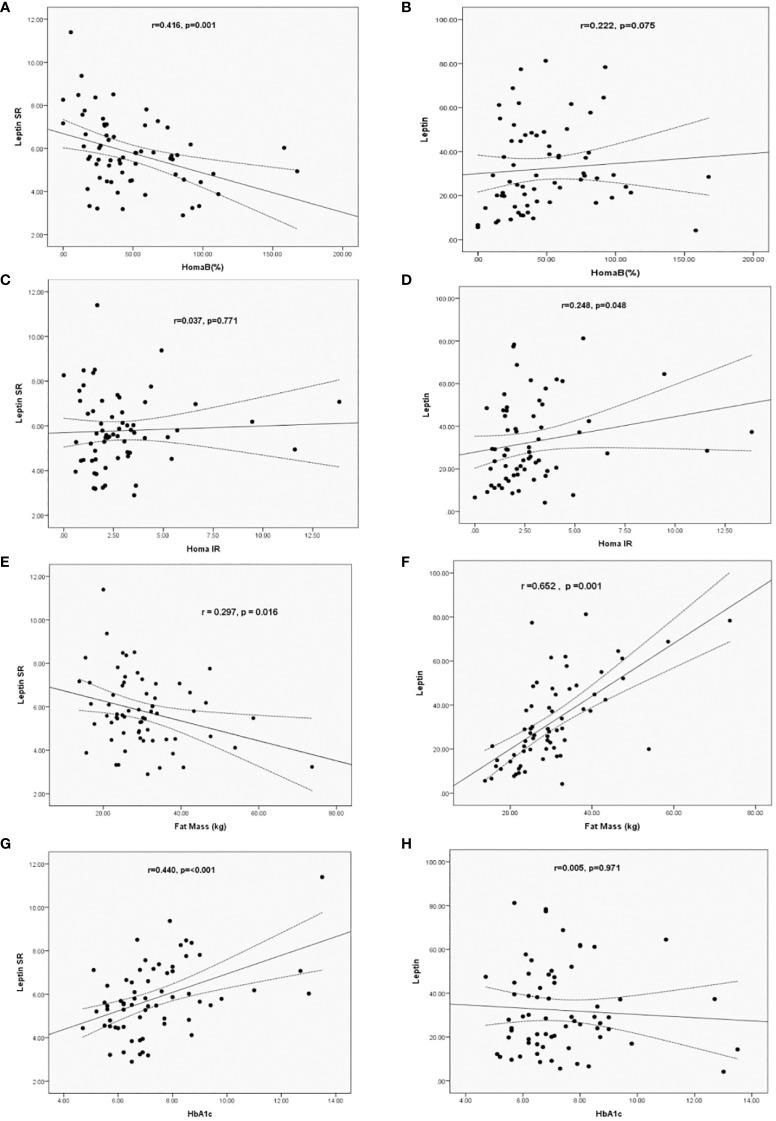
Correlation of Leptin SR **(A, C, E, G)** and leptin **(B, D, F, H)** levels with HOMA B, HOMA-IR, HbA1c and Fat Mass in patients with T2DM.

Using simple regression analysis we determined the correlations between levels of leptin and leptin SR with BFM and HOMA-IR, HOMA-B and HbA1c in patients with T2DM. Leptin SR correlated negatively with HOMA-B (r= -0.416, p=0.001), while the correlation of leptin with HOMA-B was non-significant (r=0.222, p=0.075) (**Figures 1A, B**). Leptin correlated significantly with HOMA-IR (r=0.248, p=0.048) while the correlation of leptin SR with HOMA-IR was not significant (r=0.037, p=0.771) (**Figures 1C, D**).

Leptin SR had a significant negative correlation (r= -0.297, p=0.016) with fat mass which was positively correlated with leptin level (r=0.652, p=0.001) (**Figures 1E, F**). Significant positive correlation between HbA1c and leptin SR was observed (r=0.440, p=<0.001), while the correlation was not significant with leptin levels (r=0.005, p=0.971) (**Figures 1G, H**).

A multivariate regressions analysis model was constructed to find significant predictors of both serum total leptin and leptin SR among adiposity measures, insulin resistance indices and lipid markers for patients with T2DM. We adjusted for age, sex, BMI, systolic blood pressure, HbA1c, TG, HDL and LDL (**Table 2**). The independent predictors for leptin in diabetic patients were BFM (B=0.705, p<0.001) and HbA1c (B= -0.311, p=0.003) and for leptin SR were TG (B=0.325, p=0.003) and HbA1c (B=0.262, p=0.015) **Table 2**.

**Table 2:** Multivariate regression analysis for significant factors associated with serum leptin and leptin SR levels in patients with T2DM.

	B	S.E (E)	*P*
Leptin			
Body Fat Mass	0.705	0.250	0.000
HbA1c	-0.311	0.210	0.003
Leptin SR			
TG	0.325	0.207	0.003
HbA1c	0.262	0.026	0.015

In this table we showed the significant predictors only.

**Figure 2:**
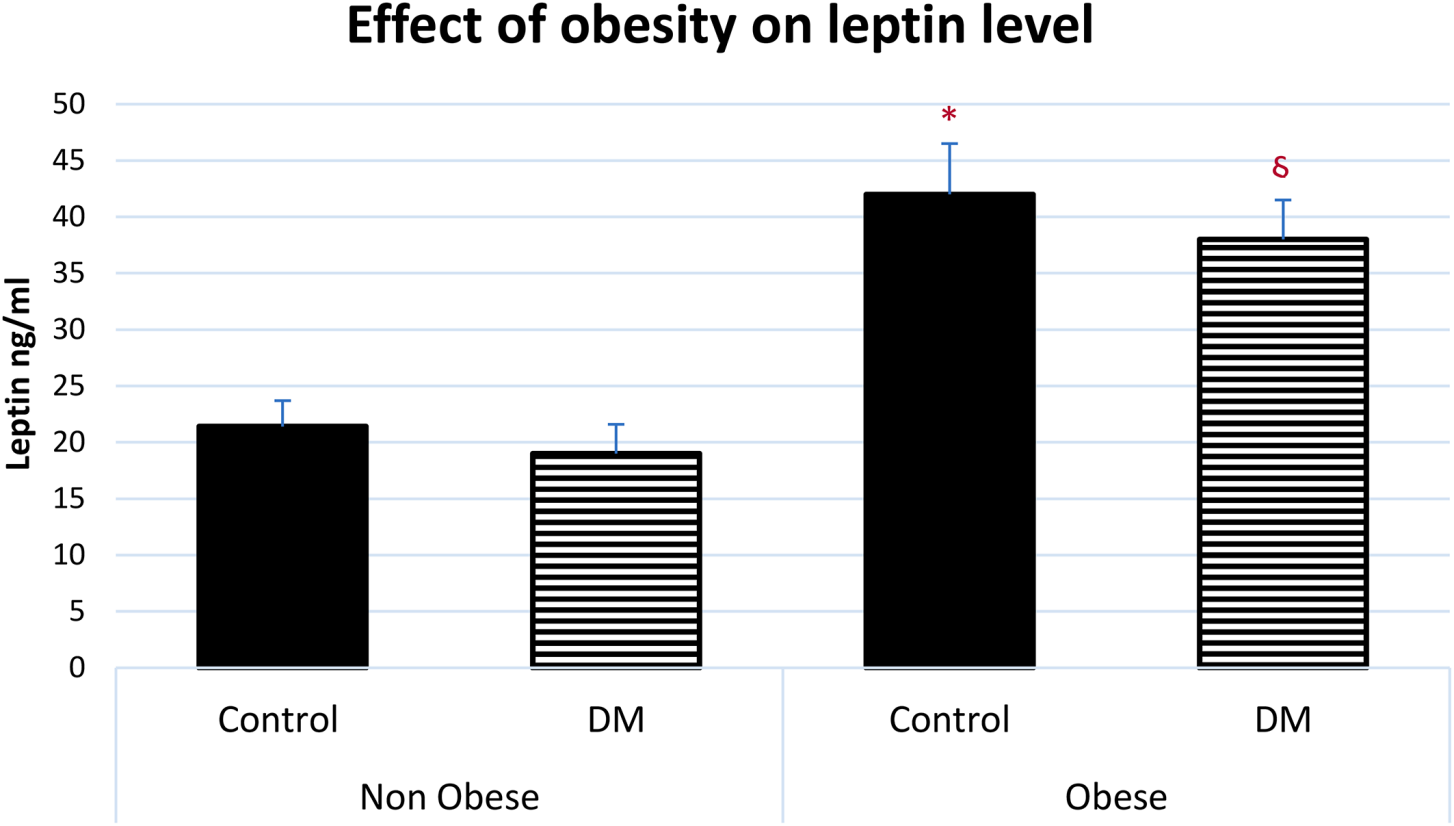
Effect of obesity on serum leptin level (mean ± SEM). Non-obese control (n=30), Non-obese diabetic (n=30), obese control (n=33), obese diabetic (n=35) * Obese control vs non-obese control, p=0.001, Obese control vs non-obese diabetic patients, p<0.0001. ^§^ Obese diabetic patients vs non-obese control, p<0.001, Obese diabetic patients vs non-obese diabetic patients, p<0.0001.

**Figure 3:**
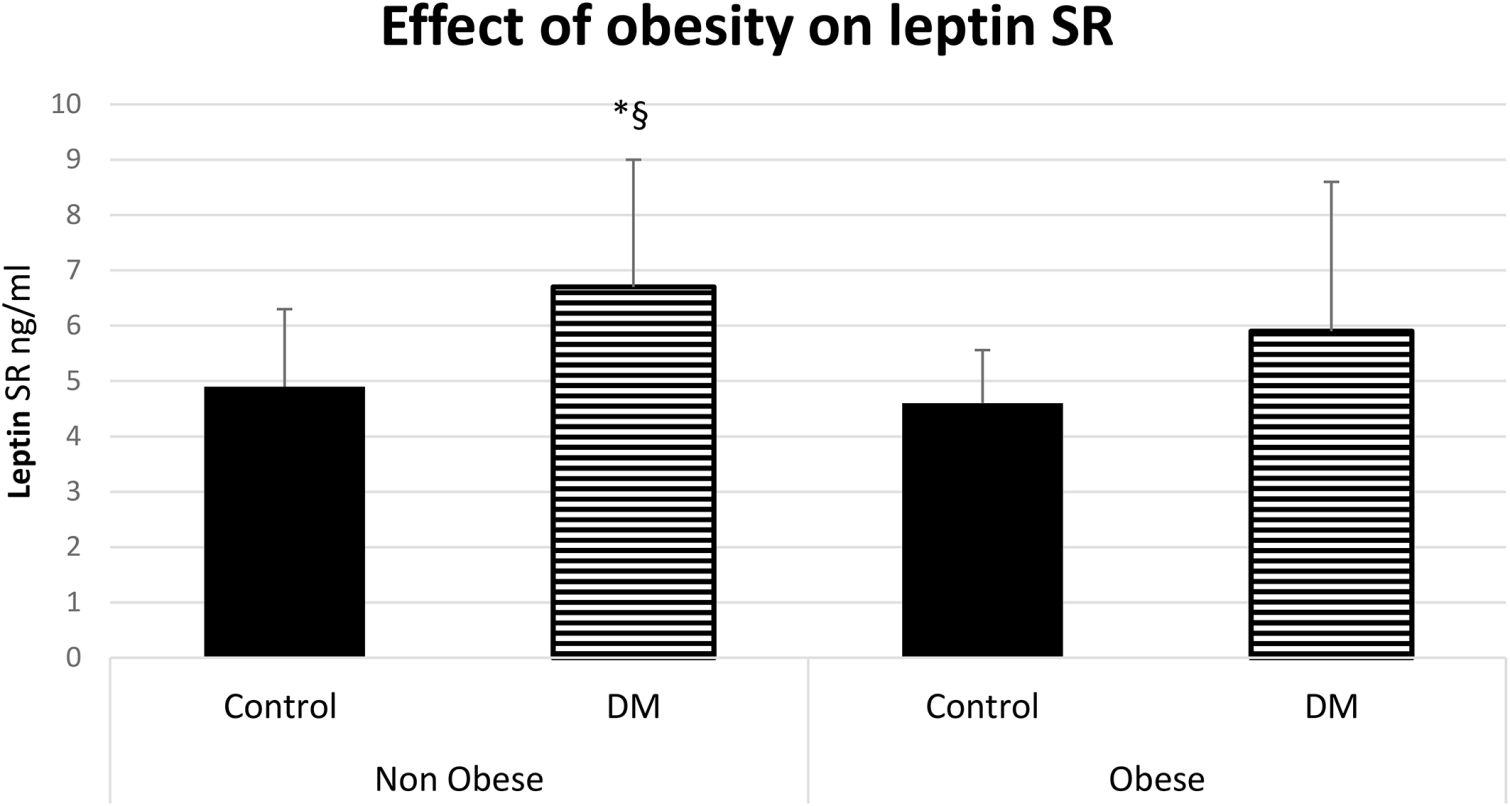
Effect of obesity on leptin soluble receptor (Leptin SR) level (mean ± SD), DM: Diabetes mellitus. Non-obese control (n=30), Non-obese diabetic (n=30), obese control (n=33), obese diabetic (n=35). ^§^ Non obese DM vs Non obese contol group (p=0.006).*Non-obese DM vs obese control group (p=0.002).

The serum level of leptin was significantly higher in obese control vs non-obese control (p=0.001) and in obese control vs non-obese diabetic patients (p<0.0001). Leptin was significantly higher in obese diabetic patients vs non-obese control (p<0.001) and in Obese diabetic patients vs non-obese diabetic patients, (p<0.0001) as shown in **Figure 2**. Leptin SR level was significantly higher in non-obese diabetic vs Non obese control group (p=0.006) and in non-obese diabetic vs obese control group (p=0.002) as shown in **Figure 3**.

**Figure 4:**
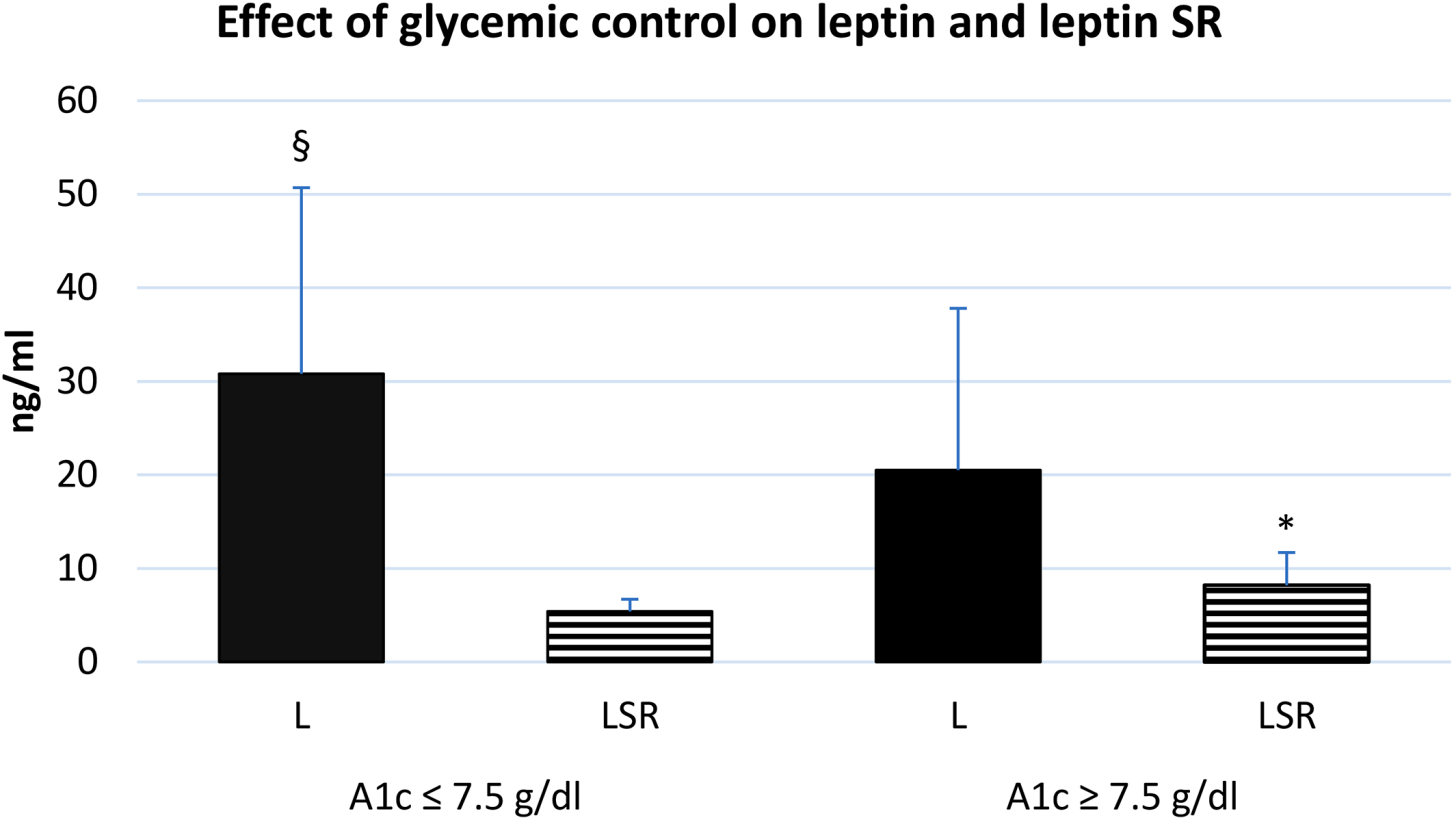
effect of glycemic control on the serum level of leptin and leptin SR. A1c: glycosylated hemoglobin A1c, L: leptin, LSR: leptin soluble receptor. Well controlled (n=35), poorly controlled (n=30). § Significant difference in L with poor glycemic control (p<0.03), * Significant difference in LSR with poor glycemic control (p<0.001).

The level of leptin and leptin SR were associated with the glycemic control, we observed that diabetic patients with poor control of blood glucose level (HbA1c >7.5) tend to have a higher serum level of leptin SR and a lower leptin level compared to patients with good control (**Figure 4**).

## Discussion

Our study demonstrated that regardless of obesity, serum leptin SR level was significantly higher in patients with T2DM compared to healthy subjects. The elevated level of leptin SR was linked to dyslipidemia specifically TG level. On the other hand, leptin level was elevated in obese subjects whether they have diabetes or not and it was correlated with BFM. A group of researchers observed that leptin level increased suddenly at BMI of 24.6 while the level of leptin SR decreased rapidly at BMI of 30. However, further increase in BMI was not associated with a further decrease in synthesis of leptin SR (11).

Interestingly leptin SR level showed a strong positive correlation with HbA1c and a significant negative association with beta cell function. Moreover, leptin SR level increases while leptin level decreases with poor glycemic control. These associations of leptin SR with glycemic indices in our patients indicate that elevated leptin SR might play a significant role in disease manifestations and severity. Data is scarce with regards to the association of leptin SR with body composition, glycemic indices and HOMA-IR in patients with T2DM (7, 12 –13). Sun and colleagues reported that independent of obesity and leptin levels, there was a strong inverse association between high levels of circulating leptin SR and the risk for development of T2DM in American women (12). In contrast, our findings suggested that increased plasma level of leptin SR in a milieu of low leptin level might play a role in pathophysiology of T2DM by causing impairment of β-cell function. One reason for low leptin SR level in Sun’s et al. study among patients with diabetes might be the higher leptin level associated with obesity in these subjects. In a previous study we reported that increased leptin level was related to obesity regardless of associated diabetes while elevated level of tumor necrosis factor-α was linked to obesity that is associated with diabetes (14).

In the current study we observed that although leptin SR was not correlated with BFM it was significantly associated with high level of TG and uncontrolled diabetes. Ogawa and coworkers’ study showed a positive correlation between leptin SR level and high density lipoprotein level (13). Another study reported that serum leptin SR contributed to carotid intima media thickness in patients with T2DM (15). Recently, Horii et al. concluded that accumulation of lipid droplets in β-cells was associated with insulin resistance which can lead to high levels of free fatty acids (FFAs) derived from degradation of triglycerides, the accumulated FFAs can flow into β-cell. In patients with T2DM due to insulin resistance, hyperglycemia combined with excess FFAs are linked to accumulation of lipid droplets in β-cell (16). Additionally, a longitudinal study which lasted for 15 years found that patients with familial combined hyperlipidemia were at high risk to develop T2DM (17). Substantially all steps in the pathway of lipotoxicity, starting with high food intake to the point of synthesis of ceramide, is protectively influenced by leptin (18). The transport of leptin was found to be inhibited by TG through direct binding with leptin or its transporter while pharmacological intervention that reduces the level of TG reversed this inhibition of leptin transport (19).Also, we observed that leptin SR level have a strong positive correlation with HbA1c value and a negative correlation with HOMA-β. Similarly, previous studies demonstrated that leptin SR had positive correlation with HbA1c in patients with type1 diabetes (20) and negative correlation with HOMA-β in patients withT2DM (8).

We postulate that the elevated leptin SR in patients with T2DM in our study played a role in leptin resistance, in addition to its role in the impairment of β-cell function. Leptin was reported to decrease β cell apoptosis and lower α cell insulin resistance which usually leads to inhibition of the pathways leading to T2DM. Lowering TG level should be an early important step in management of diabetes to protect β cells and endothelial cells from apoptosis and atherosclerosis.

In conclusion, our study showed that leptin SR is higher in patients with T2DM and is associated with abnormal β-cell function and thus it might be considered as an important marker in the pathogenesis of diabetes. Furthermore, leptin SR level is linked to HbA1c value and TG level so it can be used in monitoring the response to treatment in patients with diabetes. More studies are needed to explore the complex pathophysiological mechanisms in diabetes mellitus, and specifically focusing on investigating whether lowering TG levels would decrease leptin SR level and protect β-cell from its deleterious effect. Finding new pathways to manage T2DM will help in implementing precision medicine.

## Data availability statement

The original contributions presented in the study are included in the article/supplementary material. Further inquiries can be directed to the corresponding author.

## Ethics statement

The studies involving human participants were reviewed and approved by Institutional Review Board, College of Medicine, King Saud University, Riyadh, Saudi Arabia. The patients/participants provided their written informed consent to participate in this study.

## Author contributions

HA, literature review, data analysis, and writing manuscript. LA, idea, data collection, and manuscript revision. All authors contributed to the article and approved the submitted version.
